# Mendelian randomization analysis revealed potential metabolic causal factors for breast cancer

**DOI:** 10.1038/s41598-023-41130-4

**Published:** 2023-08-31

**Authors:** Mengshi Zhou, Mason Henricks, Valerie Loch, Gloria Zhang, Yong Lu, Xiaoyin Li

**Affiliations:** 1 Department of Mathematics and Statistics, St. Cloud State University, 720 4th Ave S, St. Cloud, MN 56301 USA; 2 Department of Pathology, Robert J. Tomsich Pathology and Laboratory Medicine Institute, Cleveland Clinic, Cleveland, OH 44195 USA; 3 Houston Methodist Cancer Center/Weill Cornell Medicine, Houston, TX 77030 USA

**Keywords:** Computational biology and bioinformatics, Cancer genetics, Cancer, Breast cancer, Cancer epidemiology

## Abstract

Observational studies showed that metabolic phenotypes were associated with the risk of developing breast cancer (BC). However, those results are inconsistent regarding the magnitude of the association, particularly by subtypes of breast cancer. Furthermore, the mechanisms of the association remain unclear. We performed two-sample Mendelian randomization (MR) analyses to evaluate the causal effect of metabolic risk factors on breast cancer in the European population. Assessed individually using MR, body mass index (BMI) (odds ratio [OR] 0.94, 95% Confidence interval [CI] 0.90–0.98, *P* = 0.007), high-density lipoprotein cholesterol (HDL-C) (OR 1.10, 95% CI 1.07–1.13, *P* = 6.10 × 10^–11^) and triglycerides (TG) (OR 0.92, 95% CI 0.90–0.96, *P* = 1.58 × 10^–6^) were causally related to breast cancer risk. In multivariable MR, only HDL-C (OR 1.08; 95% CI 1.02–1.14; *P* = 0.02) retained a robust effect, suggesting that the genetic association between BMI, HDL-C and TG with breast cancer risk in univariable analysis was explained via HDL-C. These findings suggest a possible causal role of HDL-C in breast cancer etiology.

## Introduction

Breast cancer (BC) is the most prevalent invasive cancer and the second leading cause of cancer death in women. The National Cancer Institute and the Centers for Disease Control and Prevention claim that breast cancer risk was 129.7 per 100,000 women in the US in 2019 (95% Confidence interval [CI] 129.2–130.2). The mortality rate was 19.4 per every 100,000 women (95% CI 19.2–19.6). The burden of breast cancer motivates scientists and researchers to comprehend better the etiology of breast cancer and provide more effective prevention strategies.

While the exact cause of breast cancer is not fully understood, factors such as age, genetics, and lifestyle choices have been linked to its development. There is convincing evidence that metabolic characteristics are linked to a higher risk of developing breast cancer^1–4^. For example, a common feature of many malignancies, including breast cancer, is the reprogramming of lipid metabolism^5,6^. The mortality rate among women diagnosed with breast cancer has been associated with type 2 diabetes (T2D)^7^. Breast cancer frequently occurs with a set of metabolic risk factors including diabetes, central obesity, and poor lipid profiles. Those observational studies indicated that those risk variables are all likely to be regulated by genetic and environmental risk factors^4,5,8,9^.

**Figure 1:**
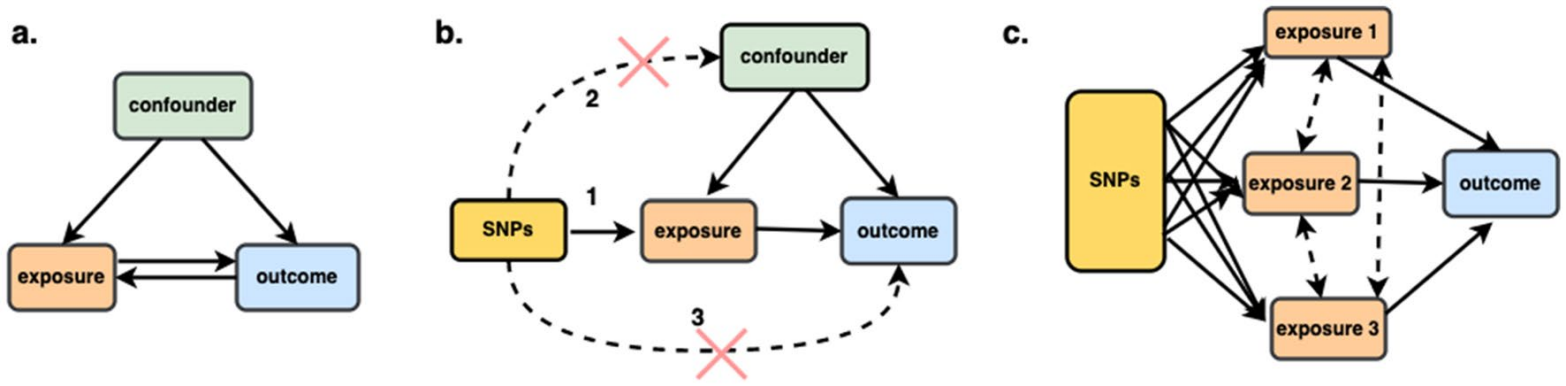
Different types of associations between exposures and outcome. (**a**) Association between exposure and outcome, may be due to confounder (**b**) Causation illustrated by Mendelian randomization with single exposure where the genetic variants (SNPs) are independent of confounding factors (**c**) Causation illustrated by Mendelian randomization with multiple correlated exposure.

However, observational analyses cannot provide evidence of causality because observational studies are subject to confounding, so it remains uncertain whether those associations are causal (Fig. 1a). Whether there is a causal link between metabolic risk factors and breast cancer is yet to be unestablished by the body of epidemiological and clinical trial data^8^. It is challenging to completely protect observational studies from bias due to reverse causation or confounding variables. Obesity has been identified as a risk factor for postmenopausal women's breast cancer^10^. However, for premenopausal women, a greater body mass index (BMI) may operate as a protective factor in reducing the incidence of breast cancer^11^. Although T2D raises the breast cancer risk, other researchers hypothesized it may actually be a marker for the adiposity-breast cancer relationship rather than the true cause of this cancer^12^. Observational epidemiological studies have found a range of associations between high-density lipoprotein cholesterol (HDL-C) and breast cancer, including positive^13^, negative^14,15^, and no relationships^16^. Mendelian randomization (MR) analysis makes it possible to evaluate the causal relationship between a particular exposure and disease risk while minimizing any biases that may be present in traditional observational studies. It is being utilized more frequently because it can overcome an important drawback of evidence from observational studies: unmeasured confounding (Fig. 1b). Using a genetic factor as an instrumental variable (IV) for the exposure, MR analysis is a method for establishing the causal relationship between an exposure and an outcome^17,18^. This method relies on the use of genetic variants as instrumental variables, typically single nucleotide polymorphisms (SNPs). It requires that (1) the genetic variants are associated with the exposure, (2) the genetic variants are independent of a measured or unmeasured confounder, and (3) the genetic variants can only influence the outcome through the exposure (Fig. 1b). Given that genetic variations are fixed at conception and are therefore unrelated to any self-adopted or environmental influences, bias present in observational study designs, such as confounding, is anticipated to be significantly reduced in MR studies^19^.

Several MR analysis techniques have been developed to determine the causal relationship between risk variables and complex diseases. The inverse variance weighted (IVW) method (with a fixed-effect meta-analysis model) is most effective when all IVs are legitimate, but it can produce biased inference when horizontal pleiotropy is presented^20^. Mendelian Randomization-Egger (MR-Egger) is one of the most used techniques that is resistant to horizontal pleiotropy but suffers from loss of power^21^. On the other hand, the weighted median technique can produce reliable casual estimates if the invalid IVs account for up to 50% of the weight of the studies^20,22^. Mendelian Randomization Pleiotropy Residual Sum and Outlier (MR-PRESSO) method is able to find outliers for preventing potential horizontal pleiotropy and correct the causal estimates^23^. The constrained maximum likelihood and model averaging (cML-MA) approach is resistant to invalid IVs as well as uncorrelated or correlated pleiotropy^24^.

Genetic markers have been used in MR research to examine the relationship between metabolic risk factors and breast cancer risk^8,25–30^. However, those studies have limitations. First, the causal effect was initially estimated using the IVW approach. The IVW requires all the genetic variants to be valid IVs, the estimation may be biased if horizontal pleiotropy is present, which is a violation of assumption 3. When IVW is used, most studies conduct sensitivity analyses such as MR-Egger or weighted median to detect or account for potential biases due to horizontal pleiotropy, the consistency between the estimates from the IVW analyses and sensitivity analyses should be assessed. Studies have shown that complex diseases frequently exhibit horizontal pleiotropy, which can lead to severe bias in MR settings^23^. Second, the causal relationships between metabolic risk factors and breast cancer were investigated separately. Evidence indicated that these exposures were closely related, which makes it more difficult to determine whether one exposure mediates another exposure, or whether both exposures have a simultaneous causal effect on the outcome^31^ (Fig. 1c). Third, most of the studies focused on breast cancer risk, but only a few studies were conducted on breast cancer subtypes, such as estrogen receptor (ER)-positive and ER-negative^28–30^.

In this study, we sought to identify which metabolic risk factors account for a causal relationship with breast cancer risk from a human genetics’ perspective. First, we conducted two-sample mendelian randomization analyses to assess the relationship between each of the six metabolic risk factors and breast cancer risk separately. In order to accurately evaluate the causal relationship, we applied a cutting-edge approach, called the constrained maximum likelihood and model averaging (cML-MA) method. The cML-MA method produces a robust estimate by eliminating invalid IVs (i.e. pleiotropic variants)^24^. Second, we used the multivariable MR, which estimates the causal effects of multiple exposures on breast cancer risk simultaneously. Multivariable MR helps to elucidate which of the metabolic traits is of fundamental relevance to breast cancer (Fig. 1c). Thirdly, we analyzed the two breast cancer subtypes ER-positive (ER +) and ER-negative (ER −). Lastly, we preformed sex-specific Mendelian randomization analysis with betas from genome-wide association study (GWAS) of the metabolic risk factors conducted among women of European ancestry or predominantly European ancestry. Our results provide a better understanding of the metabolic mechanisms driving breast cancer.

## Results

### Univariable Mendelian randomization analysis

**Figure 2:**
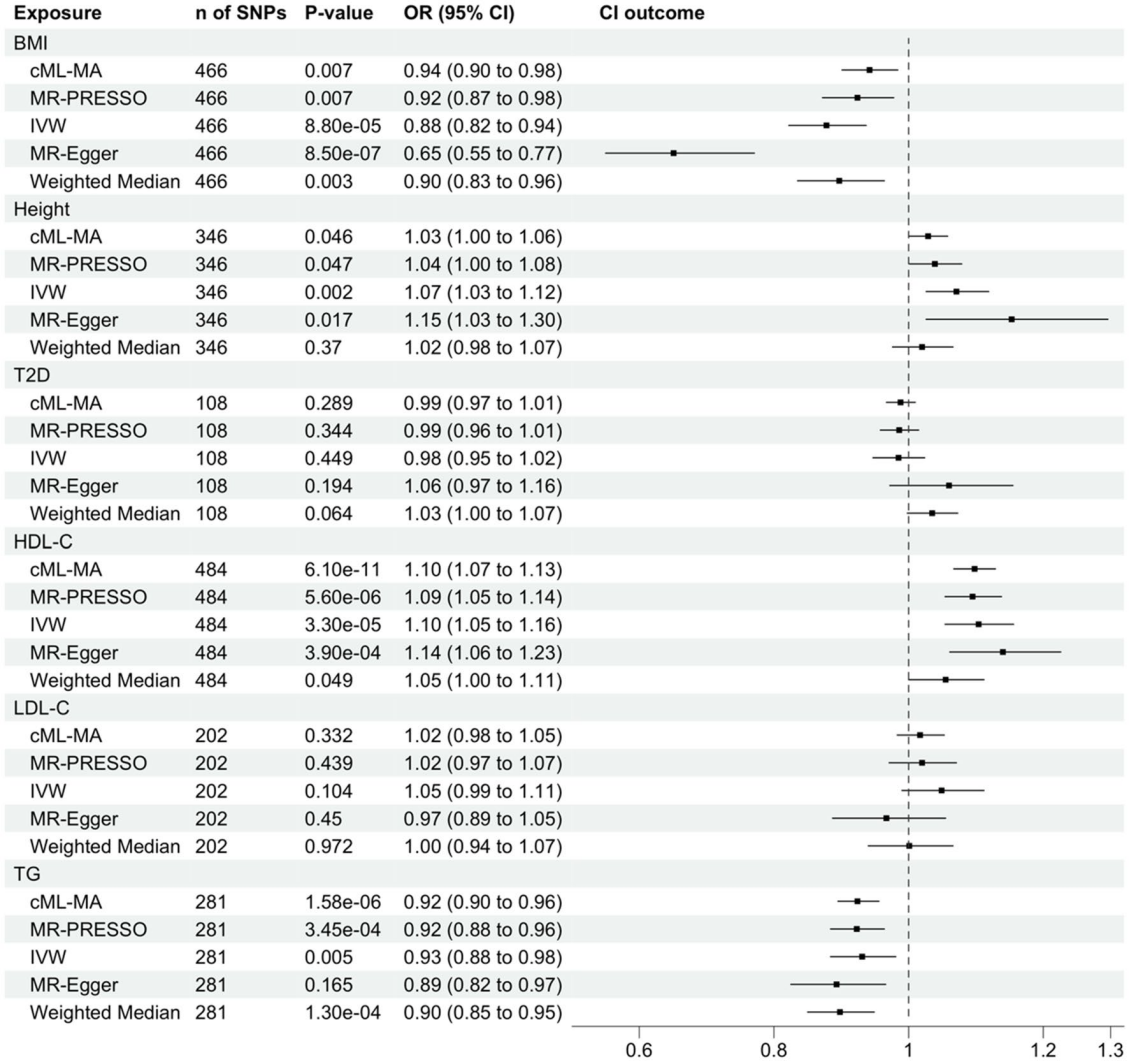
Univariable Mendelian randomization models assessing the causal effects of six metabolic risk factors on breast cancer risk. SNP, single nucleotide polymorphisms; *P*-value, corresponding *P*-value; OR, odds ratio; CI, confidence interval; BMI, Body Mass Index; cML-MA, the constrained maximum likelihood and model averaging; MR-PRESSO, Mendelian Randomization Pleiotropy Residual Sum and Outlier; IVW, Inverse Variance Weighted; MR-Egger, Mendelian Randomization-Egger; T2D, Type 2 Diabetes; HDL-C, High-Density Lipoprotein Cholesterol; LDL-C, Low-Density Lipoprotein Cholesterol; TG, triglycerides.

On individual assessment through MR analysis, body mass index was found to be a protective factor against breast cancer. A 1-standard-deviation–higher BMI had an odds ratio (OR) of 0.94 (95% CI 0.90–0.98, *P* = 0.007) for breast cancer (Fig. 2 and Table S1). To evaluate the robustness of the associations, we conducted four additional MR analyses which served as sensitivity analyses. This negative association was consistent with the MR-PRESSO, IVW, MR-Egger, and Weighted Median methods. All analyses yielded similar results, except MR-Egger with an estimate of 0.65. That may be due to the presence of horizontal pleiotropy. Using cML-MA, 16 SNPs were identified as potential pleiotropy outliers (Figure S1a, Figure S4a, and Table S9). The data was also displayed visually by a funnel plot. In the context of MR, asymmetry in the funnel plot will occur if there is directional pleiotropy; that is, whether causal estimates from weaker variants tend to be skewed in one direction^21^. The funnel plot is symmetric after excluding the 16 pleiotropy outliers (Figure S4a), which indicates that the horizontal pleiotropy effect has been removed using cML-MA. There was no evidence of week instrument bias (all F-Statistic > 10) (Table S3), with little evidence of heterogeneity by both the Q test (Q = 819.0, *P* < 0.05) and the I^2^ statistic (I^2^ > 75%) (Table S10). We observed that the overall causal effects for breast cancer were not driven by any individual SNP from the leave-one-out analysis (Figure S7a).

For HDL-C, a one standard deviation increment in HDL-C, gave an odds ratio for breast cancer of 1.10 (95% CI 1.07–1.13, P = 6.1 × 10^–11^) (Fig. 2 and Table S1). 19 outliers have been detected and excluded in the cML-MA analysis, which indicates evidence of horizontal pleiotropy (Figure S1d, Figure S4d, and Table S9). After removing the pleiotropy outliers, the funnel plot was symmetrical (Figure S4d). The association between HDL-C and breast cancer was also consistent with the MR-PRESSO, IVW, and MR-Egger methods. All SNPs have an F-Statistic > 10, which suggests no evidence of week instrument bias (Table S6). Lastly, the leave-one-out analysis did not identify any influential SNPs that could affect the overall causal effects on breast cancer (Figure S7c).

TG was found to be a protective factor against breast cancer. A one standard deviation increment in TG, gave an odds ratio for BC of 0.92 (95% CI 0.90–0.96, *P* = 1.58 × 10^–6^) (Fig. 2 and Table S1). The association between TG and BC was consistent with all other MR methods. We did not observe any individual SNP affecting the overall estimate of the rest of the studies significantly from the leave-one-out analysis (Figure S7c). A formal assessment revealed a minimal risk of bias from week instrument variables with all F statistic > 10 (Table S8).

### Multivariable Mendelian randomization analysis

**Figure 3:**
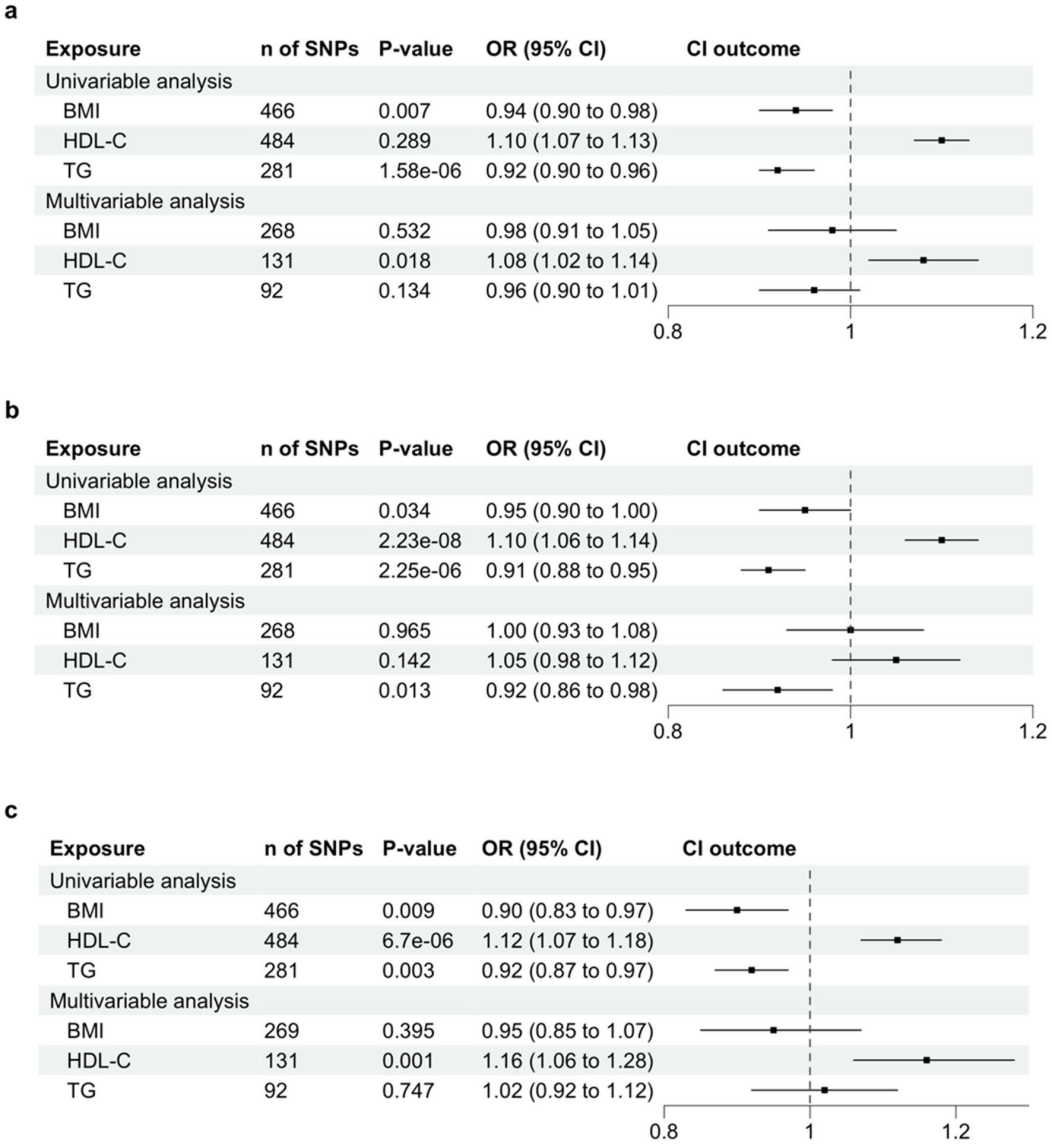
Univariable and Multivariable Mendelian Randomization Analysis of BMI, HDL-C and TG on Breast Cancer Risk and Subtypes. (**a**) Breast Cancer (**b**) Estrogen receptor (ER) positive (**c**) Estrogen receptor (ER) negative. SNP, single nucleotide polymorphisms; *P*-value, corresponding *P*-value; OR, odds ratio; CI, confidence interval; BMI, Body Mass Index; HDL-C, High-Density Lipoprotein Cholesterol; TG, triglycerides.

When BMI, HDL-C and TG were assessed together in multivariable MR, only the causal relationship between HDL-C and BC risk remained robust (Fig. 3a). The adjusted OR for HDL-C with BC risk was 1.08 (95% CI 1.02–1.14; *P* = 0.018). The causal estimate of both BMI and TG was diminished. The adjusted OR for BMI is 0.98 (95% CI 0.91–1.05; *P* = 0.532), and for TG is 0.96 (95% CI 0.90–1.01; *P* = 0.134). Both P-values were not significant, suggesting the causal estimates were not distinguishable from the null. The multivariable MR results indicated that there was robust evidence for a potential causal role of HDL-C in the development of BC after adjusting for BMI and TG.

### MR analysis with outcome stratified by ER status

Next, we performed the MR analysis after stratifying the breast cancer by ER + and ER- status. The six metabolic-related traits' effect size estimates on the BC subtypes were comparable to those on the overall risk for breast cancer (Table S1, Figure S2 and S3). BMI was discovered to be both substantially associated with ER + (OR 0.946, 95% CI 0.898–0.996, *P* = 0.034) and with ER- (OR 0.901, 95% CI 0.834–0.974, *P* = 0.009). Height is found only to be significantly related to ER + (OR 1.074, 95% CI 1.04–1.109, *P* = 1.31 × 10^–5^). There is compelling evidence that HDL-C is positively correlated with both ER + (OR 1.099, 95% CI 1.063–1.136, *P* = 2.23 × 10^–8^) and ER- (OR 1.123, 95% CI 1.067–1.180, *P* = 6.70 × 10^–6^). TG is also found to be negatively correlated with both ER + (OR 0.914, 95% CI 0.880–0.949, *P* = 2.25 × 10^–6^) and ER − (OR 0.917, 95% CI 0.866–0.971, *P* = 0.003).

We observed consistent results in the causal estimates when we utilized ER + or ER- as the outcome in multivariable MR (Fig. 3b and c). Interestingly, When BMI, HDL-C and TG were assessed together in multivariable MR for ER + , only the causal relationship between TG and BC risk remained robust (the adjusted OR 0.92, 95% CI 0.86–0.98, *P* = 0.013) (Fig. 3b). For ER-, The association between HDL-C and ER- remained robust. The adjusted OR for HDL-C with ER- was 1.16 (95% CI 1.06–1.28; *P* = 0.001) (Fig. 3b).

### Sex-specific Mendelian randomization analysis

**Figure 4:**
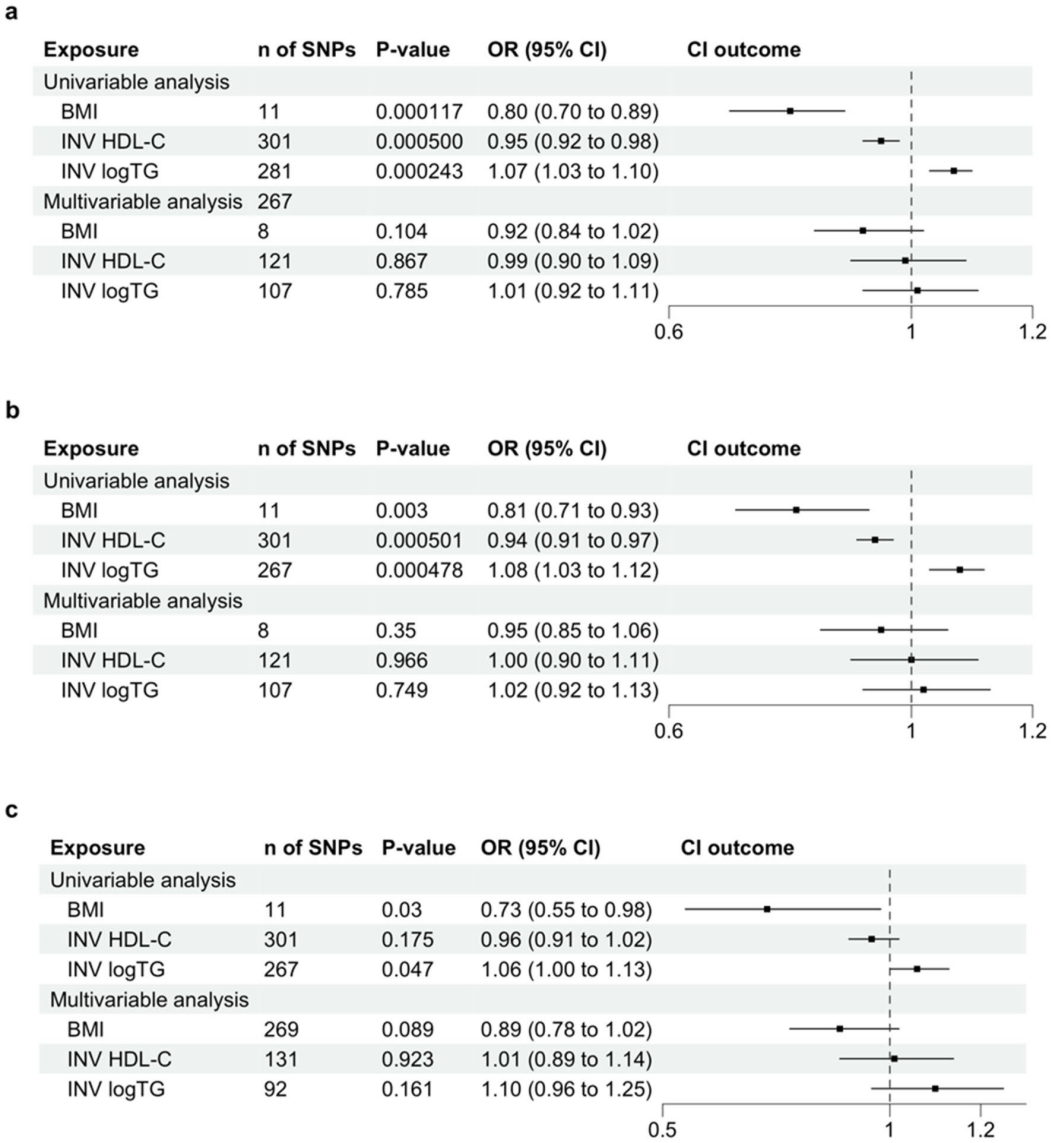
Univariable and Multivariable Mendelian Randomization Analysis of BMI, INV HDL-C and INV logTG on Breast Cancer Risk and Subtypes Using Sex-specific GWAS Studies. (**a**) Breast Cancer (**b**) Estrogen receptor (ER) positive (**c**) Estrogen receptor (ER) negative. Abbreviations: SNP, single nucleotide polymorphisms; *P*-value, corresponding *P*-value; OR, odds ratio; CI, confidence interval; BMI, Body Mass Index; INV HDL-C, Inverse normal transformation of High-Density Lipoprotein Cholesterol; INV logTG, Inverse normal transformation of log transformed triglycerides.

Lastly, sex-specific mendelian randomization were constructed and evaluated for the six metabolic risk factors using SNPs from GWASs conducted among women of European ancestry or predominantly European ancestry. We conducted a series of MR analysis using significantly associated SNPs in female specific GWASs from European population. We observed consistent results. BMI was discovered to be significantly associated with breast cancer risk (OR 0.80, 95% CI 0.70–0.89, *P* = 1.17 × 10^–4^) (Fig. 4a and Table S2). For the inverse normal transformation (INV) of HDL-C, a one standard deviation increment in INT HDL-C, gave an odds ratio for breast cancer of 0.95 (95% CI 0.92–0.98, *P* = 5.0 × 10^–4^) (Fig. 4a and Table S2). Association evidence was detected for inverse normal transformation of log transformed TG and BC risk (OR 1.07, 95% CI 1.03–1.10, *P* = 2.43 × 10^–4^) (Fig. 4 and Table S2). The causal estimate was consistent with the other MR methods (Table S2). Since the lipids GWASs was conducted using the rank inverse normal transformation of the phenotypes, it is expected that the sex specific MR results show opposite effects from the non sex-specific MR.

When BMI, HDL-C and TG were assessed together in multivariable MR, the causal estimate of all three traits was diminished (Fig. 4b and c). When exploring subgroup-specific effects, sample size is often limited compared to analyses in the overall sample. The estimated association effect size could be less accurate in sex-specific analyses because of a smaller sample size.

## Discussion

In this study, a two-sample MR study was used to examine the causal relationship of six metabolic risk factors with breast cancer risk. Using univariable MR analysis, we found BMI, HDL-C and TG causally linked to the breast cancer risk. When BMI, HDL-C and TG were taken into account in multivariable MR, the relationship between BMI and TG on breast cancer risk was attenuated. Only HDL-C retained a robust effect with breast cancer risk, indicating that HDL-C was responsible for the genetic association between BMI and TG with breast cancer risk in the univariable study. Our study offers empirical proof from human genetics that HDL-C is essential for metabolic risk factors to have a causal effect on breast cancer risk. These results add to our understanding of the relationships between BMI, HDL-C, TG and breast cancer and highlight the relevance of preventing high HDL-C in obese patients to reduce breast cancer risk.

Studies suggested that extra weight reduces breast cancer risk in premenopausal women^32^. However, breast cancer is rare in women before menopause^33^. That unlikely benefit is offset by the health risks of being overweight or obese including heart disease, diabetes, and an increased lifetime risk for several other cancers (https://www.cdc.gov/healthyweight/effects/index.html). Although BMI doesn't directly measure body fat, it does indicate if there is excess body fat. Body fat can influence breast cancer risk through an effect on hormones^34^. By elevating estrogen levels, having more fat tissue can raise your risk of developing breast cancer. Additionally, obese women have higher insulin levels than normal, another hormone whose elevated levels have been connected to a variety of malignancies, including breast cancer.

Our study found that high HDL-C levels may increase breast cancer risk. This result is somewhat surprising given that increasing HDL-C is thought to be healthier^28^. Higher levels of HDL cholesterol have been linked to a lower risk of heart disease. However, there’s been debate in lipid-related therapy whether targeting raised HDL-C levels is beneficial^35–37^. Our findings provide additional evidence against general use of HDL-C therapy.

The association between HDL-C and breast cancer has been reported intensively^8,13,15^. Similarly, BMI also been found to be associated with breast cancer in observational studies^2,25,26,32^. Those studies were conducted individually and did not consider the correlations among those metabolic risk factors. When analyzing those risk factors together, we observed that SNPs associated with one metabolic-related trait tend to associate with other metabolic traits, highlighting their pleiotropic nature. Therefore, assessing those metabolic risk factors together is needed. Although, in univariable analysis, we observed significant causal effects of both BMI and TG with breast cancer risk, those associations were diminished when we adjusted for HDL-C. Those findings have clarified the underlying biological pathways of BMI, HDL-C, TG and breast cancer association. Our study provided additional insight into the genetic and biological basis of the association between metabolic risk factors and breast cancer risk.

In the univariable analysis, there was evidence of horizontal pleiotropy for all the risk factors, which reflected the shared biological pathways between metabolic risk factors and breast cancer. We addressed this issue by using the cML-MA method, which removed the potential pleiotropy variants and provided a more accurate causal estimate. The pleiotropic variants identified in univariable analysis were further removed when conducting multivariable MR analysis. By taking horizontal pleiotropy into account, we have considered many of the relevant factors in our analyses. Thus, our results provided robust estimates between HDL-C and BC risk after adjusting for BMI and TG.

We performed MR analysis to explore the potential causal effects on breast cancer subtypes of six metabolic risk factors. In univariable analysis, we observed that height was only causally associated with ER + , while BMI was suggestively associated with both ER + and ER −. We found strong evidence that both HDL-C and TG causally linked to both ER + and ER- breast cancer. After multivariable analysis, only the causal effect of HDL-C remained for ER-, this is consistent with our observation of BC risk. It is interesting to note that for ER + , only the causal effect of TG remained in the multivariable MR analysis.

We did not find any significant causal relationship between T2D or LDL-C and BC risk. One possible explanation is the IVs we selected for T2D and LDL-C in our analyses are also associated with breast cancer. We noted that the funnel plots show asymmetry for both T2D and LDL-C, indicating the presence of horizontal pleiotropy. Another possibility is that the SNPs associated with T2D or LDL-C only account for a small proportion of the heritability, so there’s not enough power to detect the causal effect, as has been seen in traditional observational data.

Finding the causal link between metabolic characteristics and breast cancer risk still presents several difficulties. To continue implicating additional loci, larger sample sizes will be required, and if cohorts are pooled in the original GWAS heterogeneity among studies may become an issue. When evaluating the causal effect, horizontal pleiotropy may provide challenges. It will be required to carefully take into account the prospective IVs and statistical techniques.

## Materials and methods

### Study design

To calculate the causal inference between metabolic risk factors and breast cancer risk, this study used a two-sample MR design. The MR study required to employ three hypotheses (Fig. 1b).

- *Assumption 1* the genetic variants must be significantly associated with the exposure. SNPs that have been replicated in an independent sample and pass genome-wide significance (P < 5 × 10^–8^) are typically employed as instruments in MR investigations. The employment of weak instruments can cause MR estimates to be biased towards the confounded observational estimate. It may be beneficial to use multiple SNPs as instrument variables in MR research because common genetic variations only account for a small fraction of a trait's variance.
- *Assumption 2* the IVs ought to be independent of any confounding factors. It is technically impossible to demonstrate that this assumption is true in an MR study, but by looking at the link between the variant and known confounders of the exposure-outcome relationship, it may be possible to do so.
- *Assumption 3* there were no alternative paths by which the IVs directly impacted the outcome (breast cancer), only the exposure. This is also known as the exclusion restriction criterion or the "no pleiotropy" assumption. This presumption may be violated by horizontal pleiotropy, in which an SNP is linked to several phenotypes independently. While it is impossible to demonstrate that this assumption is true in an MR study, there are several adaptations to the standard MR design that can be utilized to identify its presence and estimate the causal effect of the exposure even when the assumption is violated.

### Data sources and instrumental variable selection

Summary statistics for all six exposures were available from public GWAS repositories (Table 1). All exposure sets included a strictly European sample. To ensure the assumption 1: the genetic variants must be significantly associated with the exposure, we selected SNPs associated with the exposure at the genome-wide significance level (*P* < 5 × 10^–8^) in the European population as instrumental variants for each exposure. For BMI, we selected 941 variants genome wide significantly associated with BMI from a GWAS from the Genetic Investigation of ANthropometric Traits (GIANT) consortium of 681,275 European individuals^38^. We selected 697 variants that were genome wide significantly associated with height from 253,288 European individuals that also came from of GWAS of the GIANT consortium^38^. For T2D, there were 139 common variants that were genome wide significantly associated with T2D from a sample of 655,666 Europeans^38^. Lastly, we selected 534 variants from a 403,943 sample of European individuals that were genome wide significantly associated with high density lipoprotein cholesterol levels, and 220 variants from a 440,546 sample of European individuals that were genome wide significantly associated with low density lipoprotein cholesterol levels ^38^. For sex-specific MR analysis, we selected sex-specific SNPs associated with the exposure at genome-wide significance level (*P* < 5 × 10^–8^) in the European population.

**Table 1:** Description of the six metabolic risk factors with available genetic data from GWAS.

Risk factor	Number of SNPs	Study reference	Sample size	Population	Year
BMI	941	Yengo et al.^38^	681,275	European	2018
BMI female	12	Randall et al.^39^	67,957	European	2013
Height	697	Wood et al.^40^	253,288	European	2014
Height female	323	Randall et al.^39^	67,957	European	2013
T2D	139	Xue et al.^41^	655,666	European	2018
T2D female	14	Morris et al.^42^	149,821	European	2012
HDL	534	Richardson et al.^43^	403,943	European	2020
HDL female	323	Kanoni et al*.*^44^	565,724	European	2022
LDL	220	Richardson et al. ^43^	440,546	European	2020
LDL female	281	Kanoni et al*.*^44^	558,498	European	2022
TG	313	Richardson et al. ^43^	441,016	European	2020
TG female	296	Kanoni et al.^44^	558,498	European	2022

Next, to ensure assumption 2, the IVs ought to be independent of any confounding factors. Unless steps are made in the MR analysis to account for any correlation structures that occur through linkage disequilibrium (LD), it is crucial to make sure the instruments chosen for an exposure are independent^45^. This was accomplished by further clumping the exposure-related SNPs in the European sample based on the linkage disequilibrium structure of the 1000 Genomes Project (r^2^ < 0.001 with any other associated SNP within 10 Megabase (Mb)).

The Breast Cancer Association Consortium (BCAC), which included 122,977 cases and 105,974 controls of European ancestry, provided effect estimates for those chosen SNPs on breast cancer risk^46^. It is possible to substitute SNPs that are LD 'proxies' if a chosen SNP was unavailable from the breast cancer GWAS study. Using data from the 1000 Genomes Project, we chose proxy SNPs that had high correlations (r^2^ > 0.8) with the desired variant.

To ensure that the effect alleles for the SNP effects in the exposure and outcome are the same, we harmonized exposure and outcome SNP effects. We flipped the sign of breast cancer effect estimates if the effect allele in the study associated with the exposure is the non-effect allele in the study associated with breast cancer. Palindromic SNPs, which have A/T or G/C alleles, can make it difficult to determine the identification of the effect allele in exposure and outcome GWASs^45^. Palindromic SNPs with an intermediate minor allele frequency (MAF > 0.42) were further eliminated^45^.

### Statistical analysis

First, we conducted two-sample mendelian randomization analyses to assess the relationship between each of the six metabolic risk factors and breast cancer risk individually. In order to accurately evaluate the causal relationship, we applied a cutting-edge approach called cML-MA which produces a robust estimate by eliminating invalid IVs (i.e. pleiotropic variants)^24^. We understand that such pruning could possibly remove variants that are in the same causal pathway with the risk of breast cancer, affecting the strength and validity of study instruments. Additionally, we applied four other MR techniques, MR-PRESSO, MR-Egger, IVW and Weighted median as sensitivity analysis. We presented results from analyses using the instruments that either included or excluded pleiotropic SNPs.

Second, individual MR analyses results were carried out to further elucidate which of the metabolic traits is of fundamental relevance to breast cancer. We further employed the multivariable MR (Fig. 1b) to examine these metabolic traits together in multivariable MR. When there are two or more correlated exposures, multivariable MR may be used to determine if all of the exposures have a causal effect on the outcome or whether one of the exposures mediates the effect of the other^31^.

The odds ratios and corresponding 95% confidence intervals for the results were displayed. To account for multiple comparisons, the Bonferroni method is used to handle the estimation of the chance of error in multiple testing for the six exposures. In the univariable MR analyses, a *P*-value between 0.008 and 0.05 was regarded as suggestively significant and a *P*-value less than 0.008 (0.05/6 after Bonferroni adjustment) as statistically significant.

### Sensitivity analysis

Besides just applying all the MR analyses described above to demonstrate sensitivity to different patterns of assumption violations, we calculated the Cochran’s Q statistic and the I^2^ statistic to quantify heterogeneity among causal estimates across all IVs. The corresponding P-value of Q statistic (*P* > 0.05) offers more reliability for causal effects^47^. The SNPs were also assessed by the F-statistics for week IV bias^48^. Additionally, we conducted a leave-one-out analysis to identify any potential high influence variant driving the causal association between metabolic traits and breast cancer risk.

## Conclusions

In conclusion, our findings demonstrated that HDL-C was critical in facilitating the causal effects of breast cancer risk. Additional MR studies of breast cancer subtypes (e.g., ER positive and ER negative) helped to establish the exact nature of this relationship. BMI, HDL-C and TG may be utilized as prognostic indicators of breast cancer and have significant clinical significance.

### Supplementary Information

Supplementary Information 1.

Supplementary Information 2.

## Data Availability

The GWAS summary datasets for BMI, height, T2D and BC are publicly available in TwoSampleMR at https://mrcieu.github.io/TwoSampleMR/. The GWAS summary datasets for HDL and LDL are available at S1 Table and S2 Table from ^43^. The Sex-specific GWAS summary datasets for BMI and height are downloaded from https://portals.broadinstitute.org/collaboration/giant/index.php/GIANT_consortium_data_files. The Sex-specific GWAS summary dataset for T2D is available at https://diagram-consortium.org/downloads.html. The Sex-specific GWAS summary datasets for HDL-C, LDL-C and TG are downloaded from https://csg.sph.umich.edu/willer/public/glgc-lipids2021/. All MR methods used in this manuscript are publicly available R packages TwoSampleMR, MendelianRandomization (https://cran.r-project.org/web/packages/MendelianRandomization/index.html), and MRcML (https://github.com/xue-hr/MRcML).
