# Supplementary material for: Mendelian randomization analysis revealed potential metabolic causal factors for breast cancer
Source: Sci Rep. 2023 Aug 31;13:14290. doi: 10.1038/s41598-023-41130-4 (PMC10471756; doi:10.1038/s41598-023-41130-4)

**Figure S1: Scatter plot of SNP effect of trait on metabolic risk factors and breast cancer risk: (a) BMI, (b) Height, (c) T2D, (d) HDL-C, (e) LDL-C, (f) TG.** Plot showing the effect sizes of the SNP effects on breast cancer (y-axes) and the SNP effects on six metabolic risk factors (x-axes) with. Each dot represents one SNP used as the genetic instrument. The slopes indicate the estimate for each of the five different MR tests. Red dots represent pleiotropy outliers detected by cML-MA method.

**
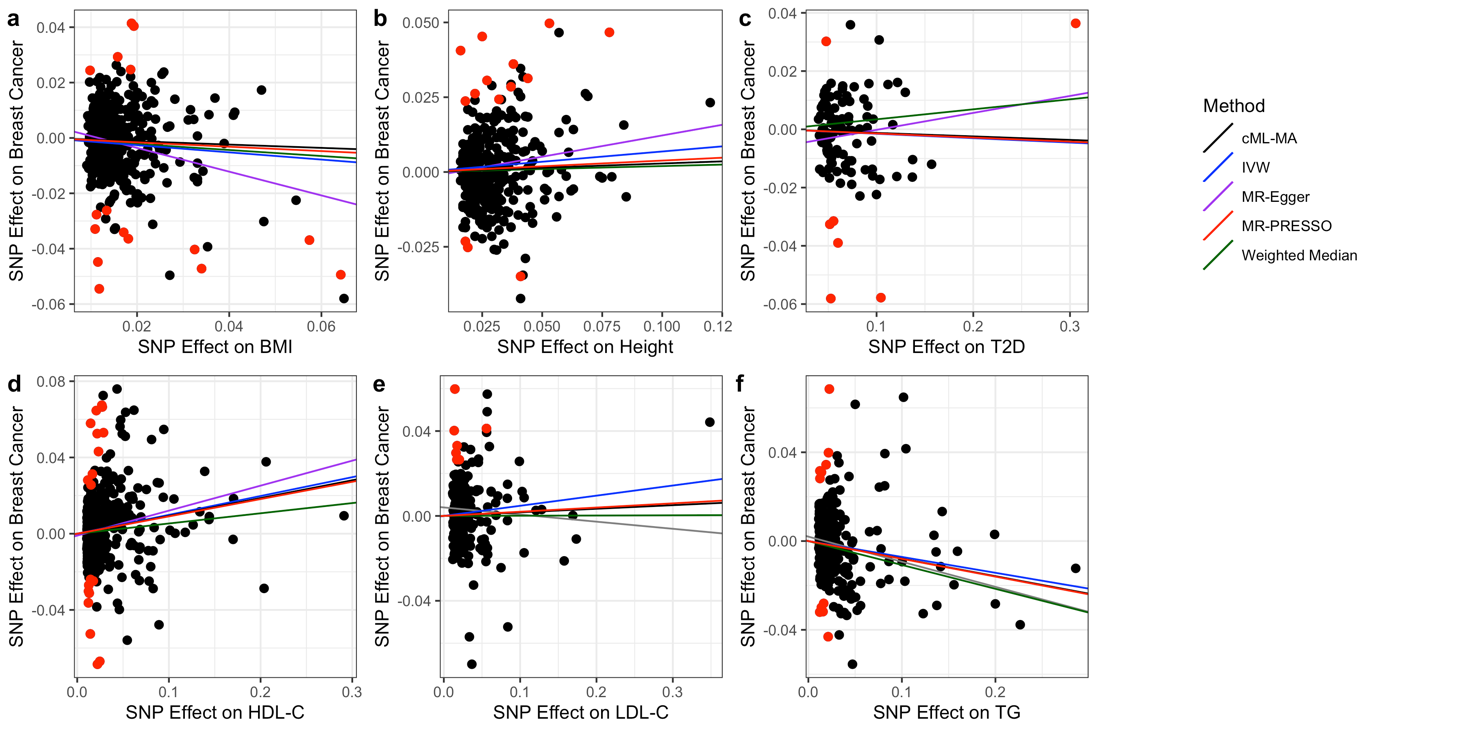
**

Abbreviations: SNP, single nucleotide polymorphisms; BMI, Body Mass Index; T2D, Type 2 Diabetes; HDL-C, High-Density Lipoprotein Cholesterol; LDL-C, Low-Density Lipoprotein Cholesterol; TG, triglycerides; cML-MA, the constrained maximum likelihood and model averaging; MR-PRESSO, Mendelian Randomization Pleiotropy Residual Sum and Outlier; IVW, Inverse Variance Weighted; MR-Egger, Mendelian Randomization-Egger.

**Figure S2: Scatter plot of SNP effect of trait on metabolic risk factors and ER-positive breast cancer: (a) BMI, (b) Height, (c) T2D, (d) HDL-C, (e) LDL-C, (f) TG.** Plot showing the effect sizes of the SNP effects on ER-positive breast cancer (y-axes) and the SNP effects on six metabolic risk factors (x-axes) with. Each dot represents one SNP used as the genetic instrument. The slopes indicate the estimate for each of the six different MR tests. Red dots represent pleiotropy outliers detected by cML-MA method.


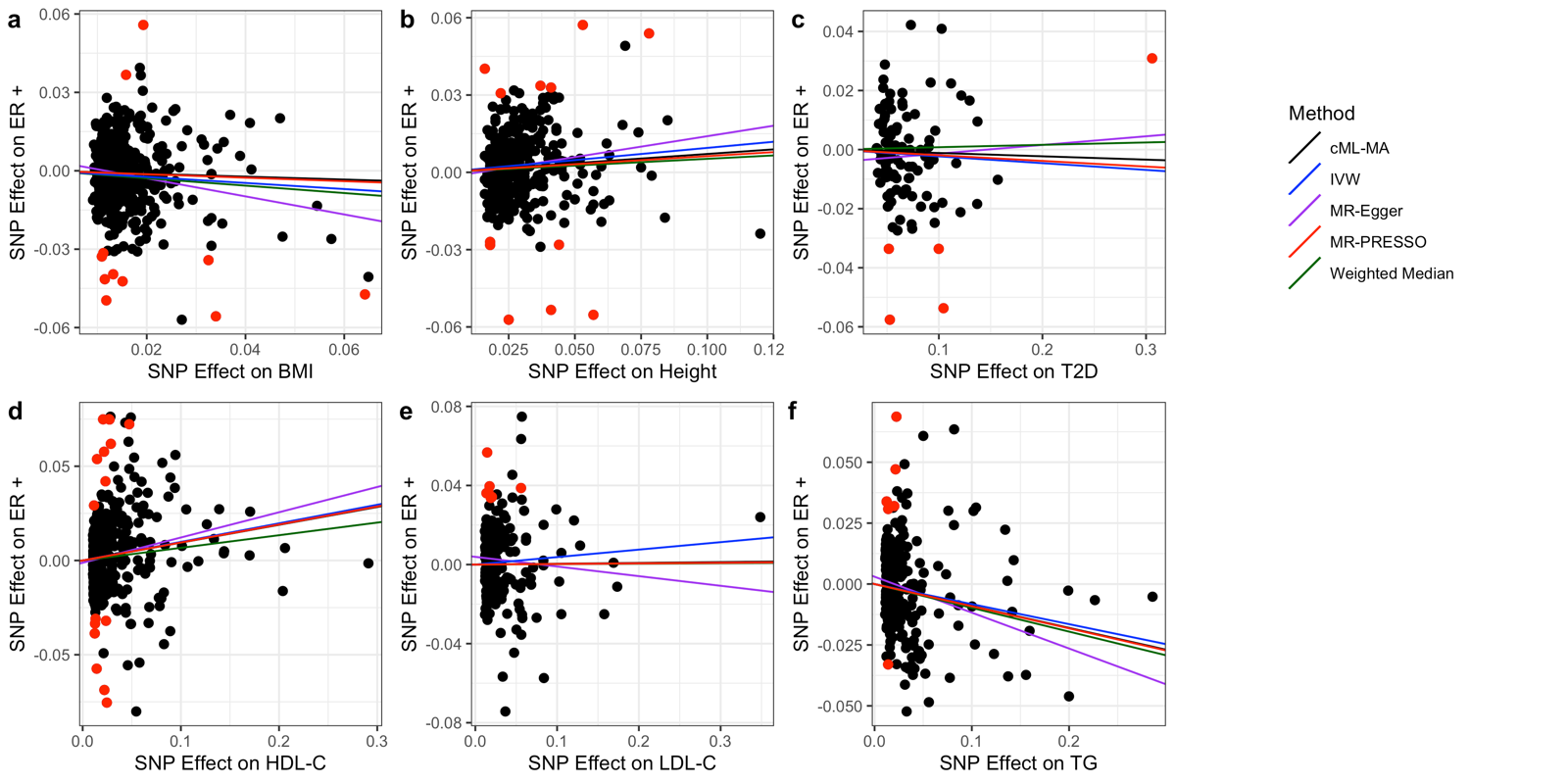


Abbreviations: SNP, single nucleotide polymorphisms; ER+, estrogen receptor positive; BMI, Body Mass Index; T2D, Type 2 Diabetes; HDL-C, High-Density Lipoprotein Cholesterol; LDL-C, Low-Density Lipoprotein Cholesterol; TG, triglycerides; cML-MA, the constrained maximum likelihood and model averaging; MR-PRESSO, Mendelian Randomization Pleiotropy Residual Sum and Outlier; IVW, Inverse Variance Weighted; MR-Egger, Mendelian Randomization-Egger.

**Figure S3: Scatter plot of SNP effect of trait on metabolic risk factors and ER-negative breast cancer: (a) BMI, (b) Height, (c) T2D, (d) HDL-C, (e) LDL-C, (f) TG.** Plot showing the effect sizes of the SNP effects on ER-negative breast cancer (y-axes) and the SNP effects on six metabolic risk factors (x-axes) with. Each dot represents one SNP used as the genetic instrument. The slopes indicate the estimate for each of the five different MR tests. Red dots represent pleiotropy outliers detected by cML-MA method.


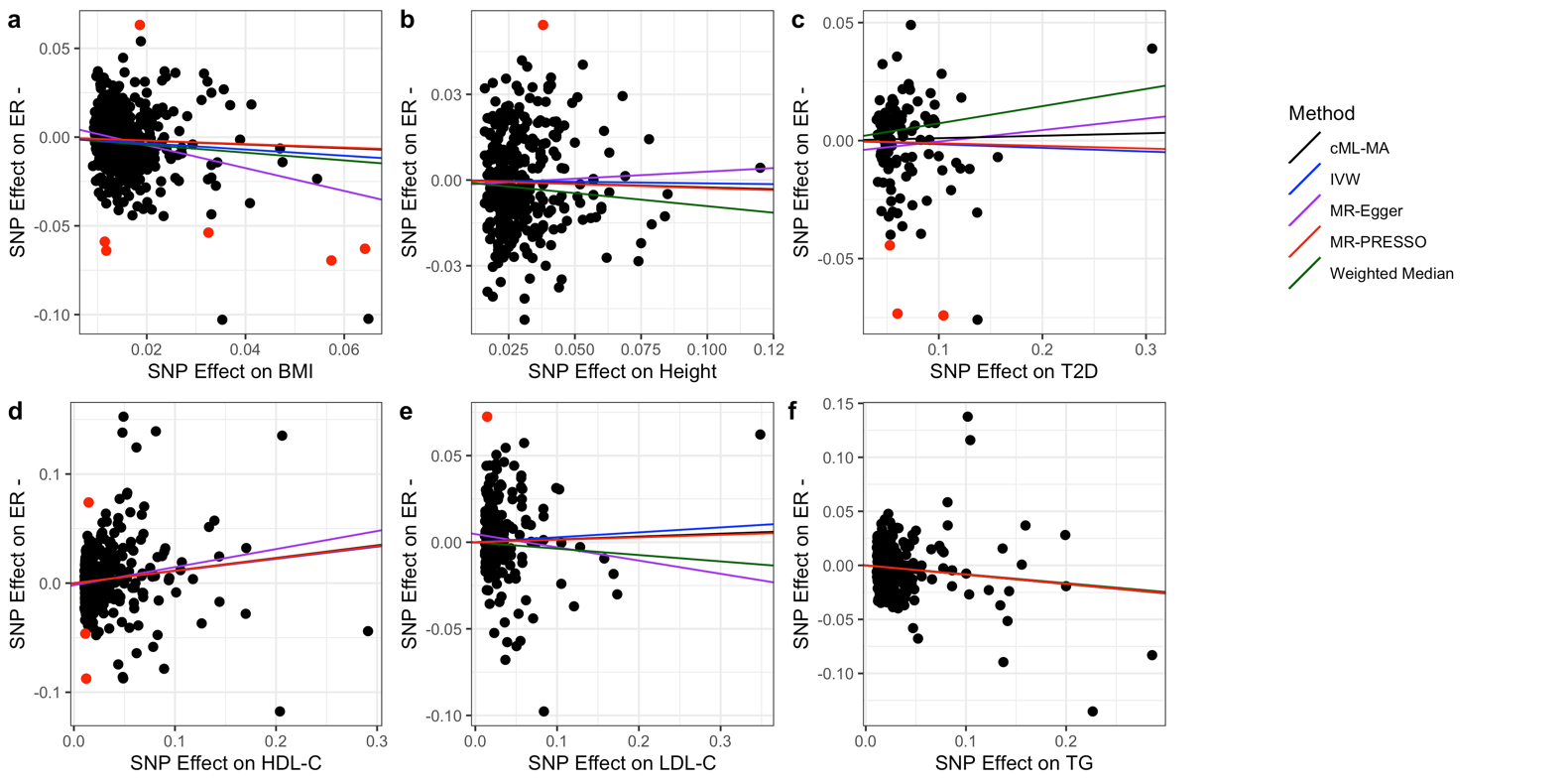


Abbreviations: SNP, single nucleotide polymorphisms; ER-, estrogen receptor negative; BMI, Body Mass Index; T2D, Type 2 Diabetes; HDL-C, High-Density Lipoprotein Cholesterol; LDL-C, Low-Density Lipoprotein Cholesterol; TG, triglycerides; cML-MA, the constrained maximum likelihood and model averaging; MR-PRESSO, Mendelian Randomization Pleiotropy Residual Sum and Outlier; IVW, Inverse Variance Weighted; MR-Egger, Mendelian Randomization-Egger.

**Figure S4: Funnel plot of six metabolic risk factors on breast cancer risk: (a) BMI, (b) Height, (c) T2D, (d) HDL-C, (e) LDL-C, (f) TG.** The plot shows the causal estimate of a particular SNP against the SNP expected precision (1/Standard Error (SE)). Asymmetry in the funnel plot is an indication of horizontal pleiotropy. The horizontal lines represent the estimate for each of the five different MR tests respectively. Red dots represent pleiotropy outliers detected by cML-MA method.


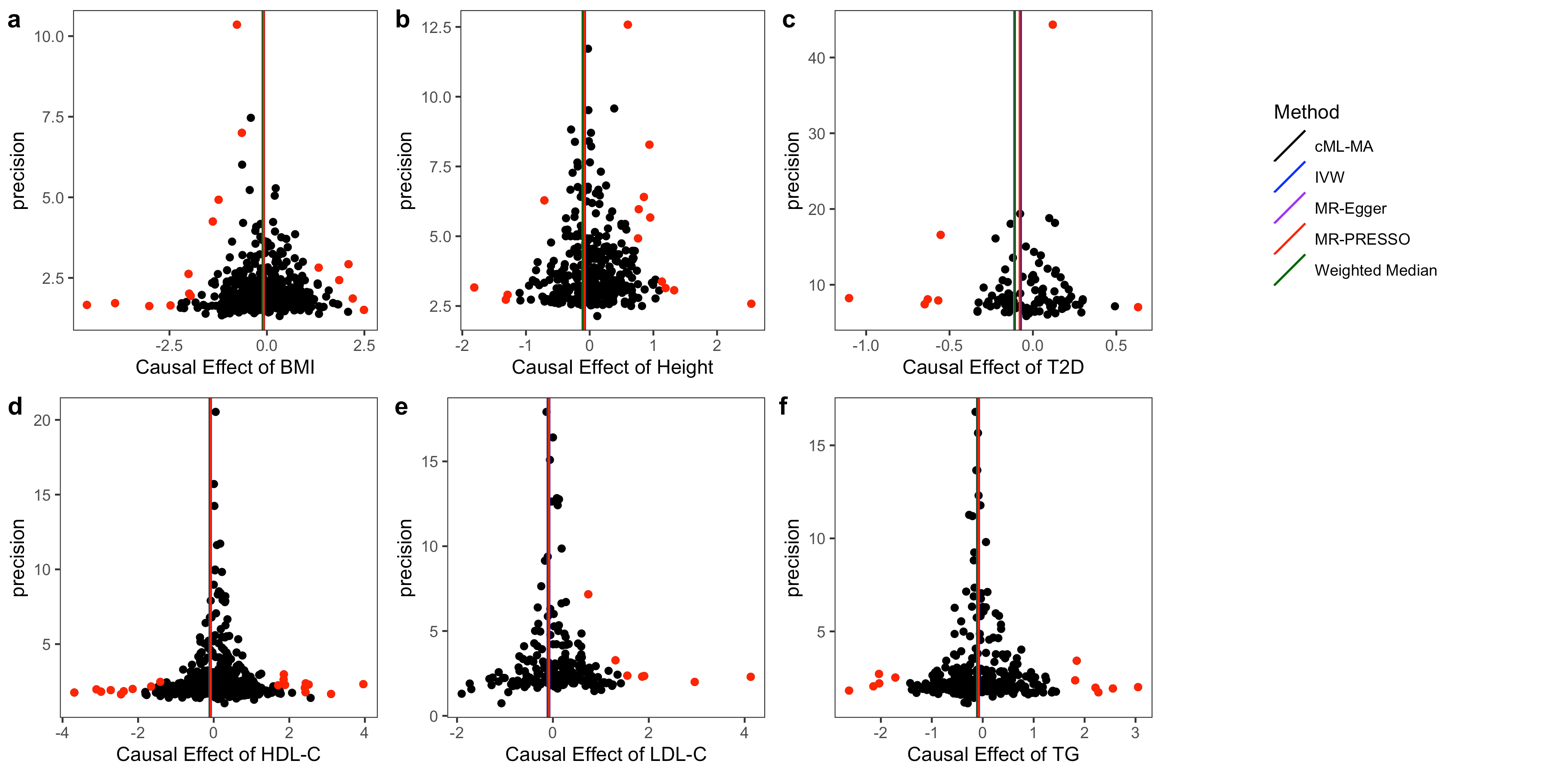


Abbreviations: SNP, single nucleotide polymorphisms; BMI, Body Mass Index; T2D, Type 2 Diabetes; HDL-C, High-Density Lipoprotein Cholesterol; LDL-C, Low-Density Lipoprotein Cholesterol; TG, triglycerides; cML-MA, the constrained maximum likelihood and model averaging; MR-PRESSO, Mendelian Randomization Pleiotropy Residual Sum and Outlier; IVW, Inverse Variance Weighted; MR-Egger, Mendelian Randomization-Egger.

**Figure S5: Funnel plot of six metabolic risk factors on ER- positive breast cancer: (a) BMI, (b) Height, (c) T2D, (d) HDL-C, (e) LDL-C, (f) TG.** The plot shows the causal estimate of a particular SNP against the SNP expected precision (1/Standard Error (SE)). Asymmetry in the funnel plot is an indication of horizontal pleiotropy. The horizontal lines represent the estimate for each of the five different MR tests respectively. Red dots represent pleiotropy outliers detected by cML-MA method.
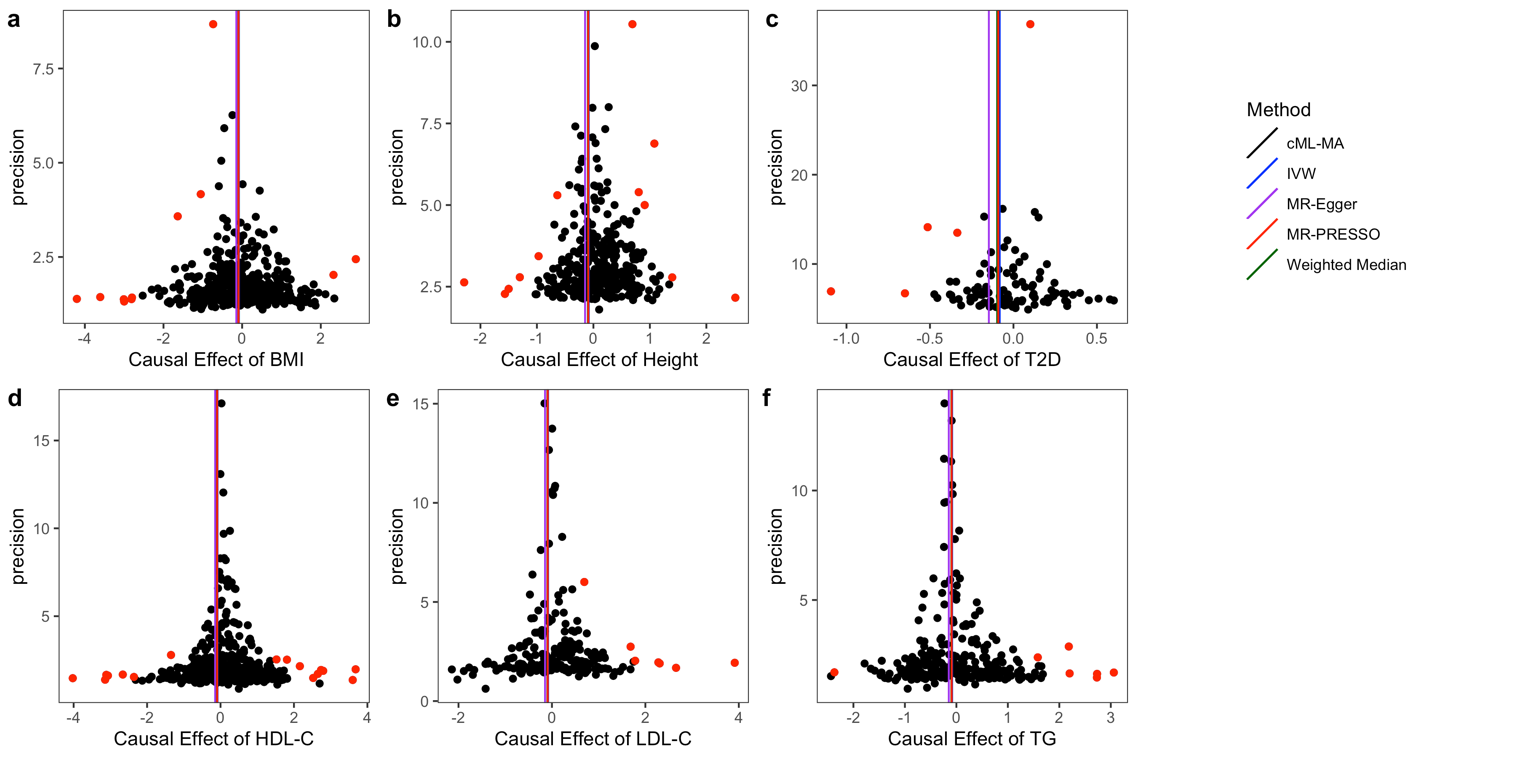


Abbreviations: SNP, single nucleotide polymorphisms; ER+, estrogen receptor positive; BMI, Body Mass Index; T2D, Type 2 Diabetes; HDL-C, High-Density Lipoprotein Cholesterol; LDL-C, Low-Density Lipoprotein Cholesterol; TG, triglycerides; cML-MA, the constrained maximum likelihood and model averaging; MR-PRESSO, Mendelian Randomization Pleiotropy Residual Sum and Outlier; IVW, Inverse Variance Weighted; MR-Egger, Mendelian Randomization-Egger.

**Figure S6: Funnel plot of six metabolic risk factors on ER- negative breast cancer: (a) BMI, (b) Height, (c) T2D, (d) HDL-C, (e) LDL-C, (f) TG.** The plot shows the causal estimate of a particular SNP against the SNP expected precision (1/Standard Error (SE)). Asymmetry in the funnel plot is an indication of horizontal pleiotropy. The horizontal lines represent the estimate for each of the five different MR tests respectively. Red dots represent pleiotropy outliers detected by cML-MA method.


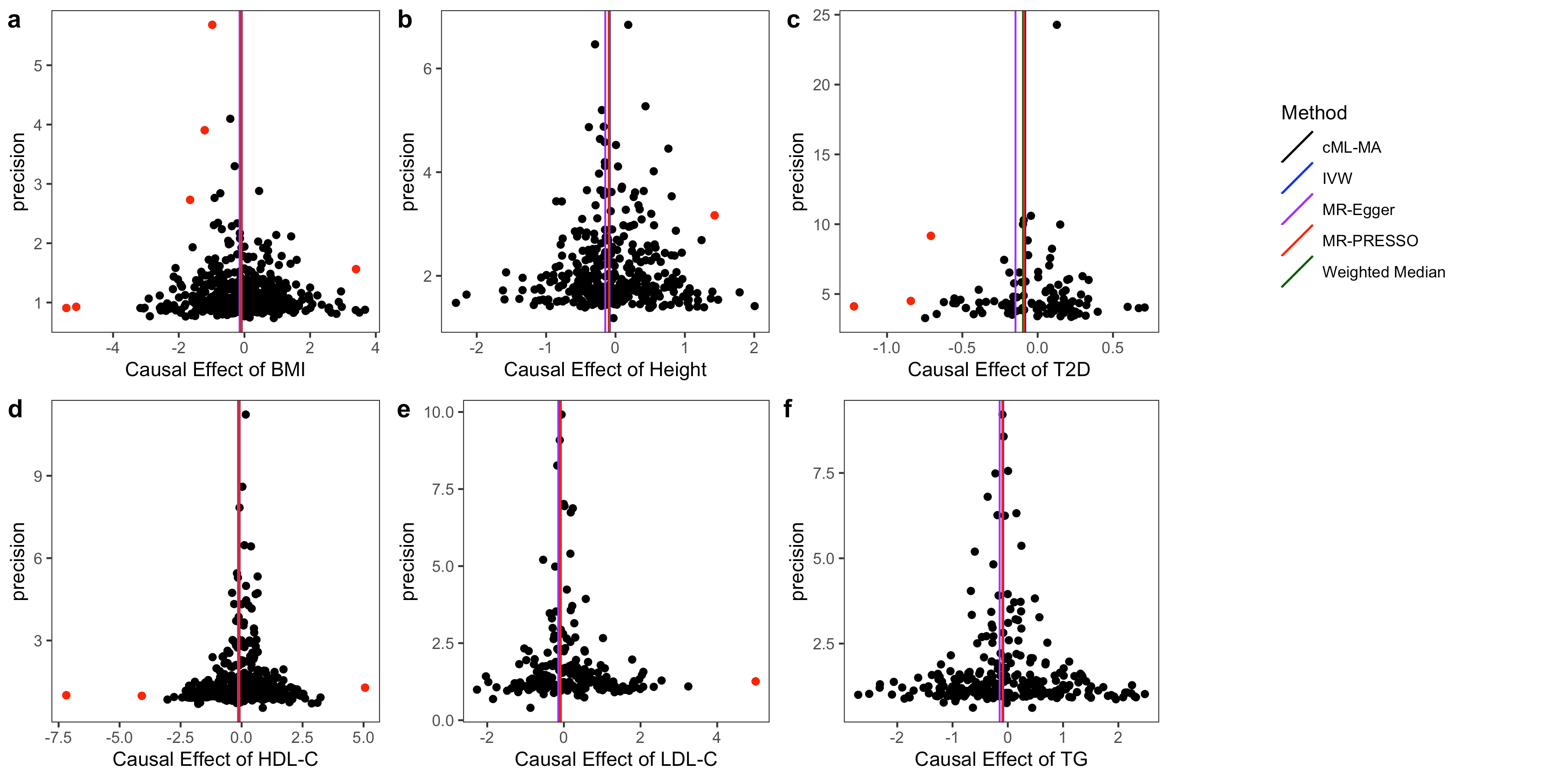


Abbreviations: SNP, single nucleotide polymorphisms; ER-, estrogen receptor negative; BMI, Body Mass Index; T2D, Type 2 Diabetes; HDL-C, High-Density Lipoprotein Cholesterol; LDL-C, Low-Density Lipoprotein Cholesterol; TG, triglycerides; cML-MA, the constrained maximum likelihood and model averaging; MR-PRESSO, Mendelian Randomization Pleiotropy Residual Sum and Outlier; IVW, Inverse Variance Weighted; MR-Egger, Mendelian Randomization-Egger.

**Figure S7: Leave-one-out plot for BMI, HDL-C and TG with breast cancer risk: (a) Height, (b) HDL-C, (c) TG.** Plot shows the estimate effect by sequentially dropping one SNP at a time. Each black dot in the forest plot represents the MR results (IVW method) excluding that particular SNP. The result including all SNPs is shown in red at the bottom of the plot.


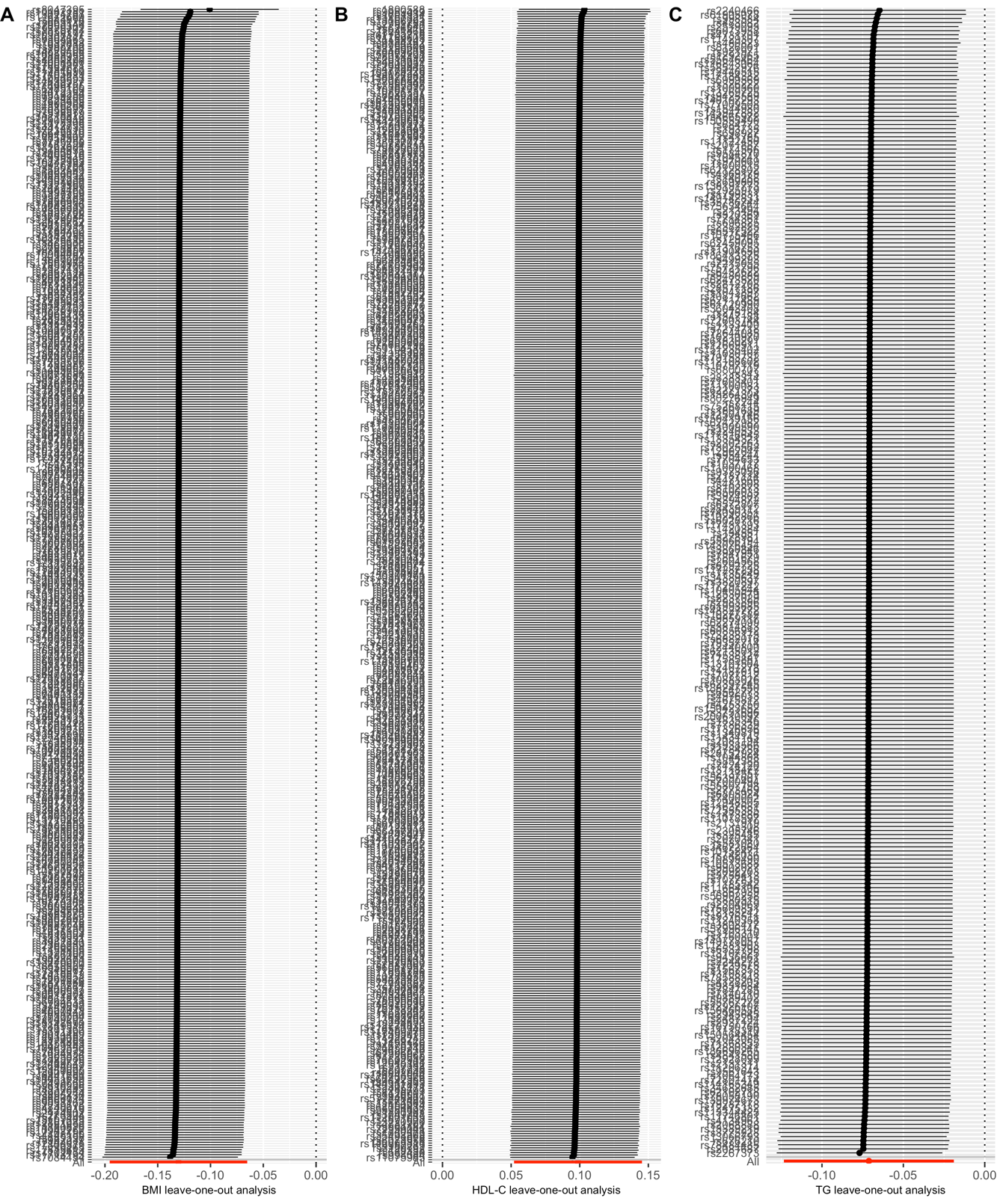


**Figure S8: Leave-one-out plot for BMI, HDL-C and TG with ER-positive breast cancer: ((a) Height, (b) HDL-C, (c) TG.** Plot shows the estimate effect by sequentially dropping one SNP at a time. Each black dot in the forest plot represents the MR results (IVW method) excluding that particular SNP. The result including all SNPs is shown in red at the bottom of the plot.


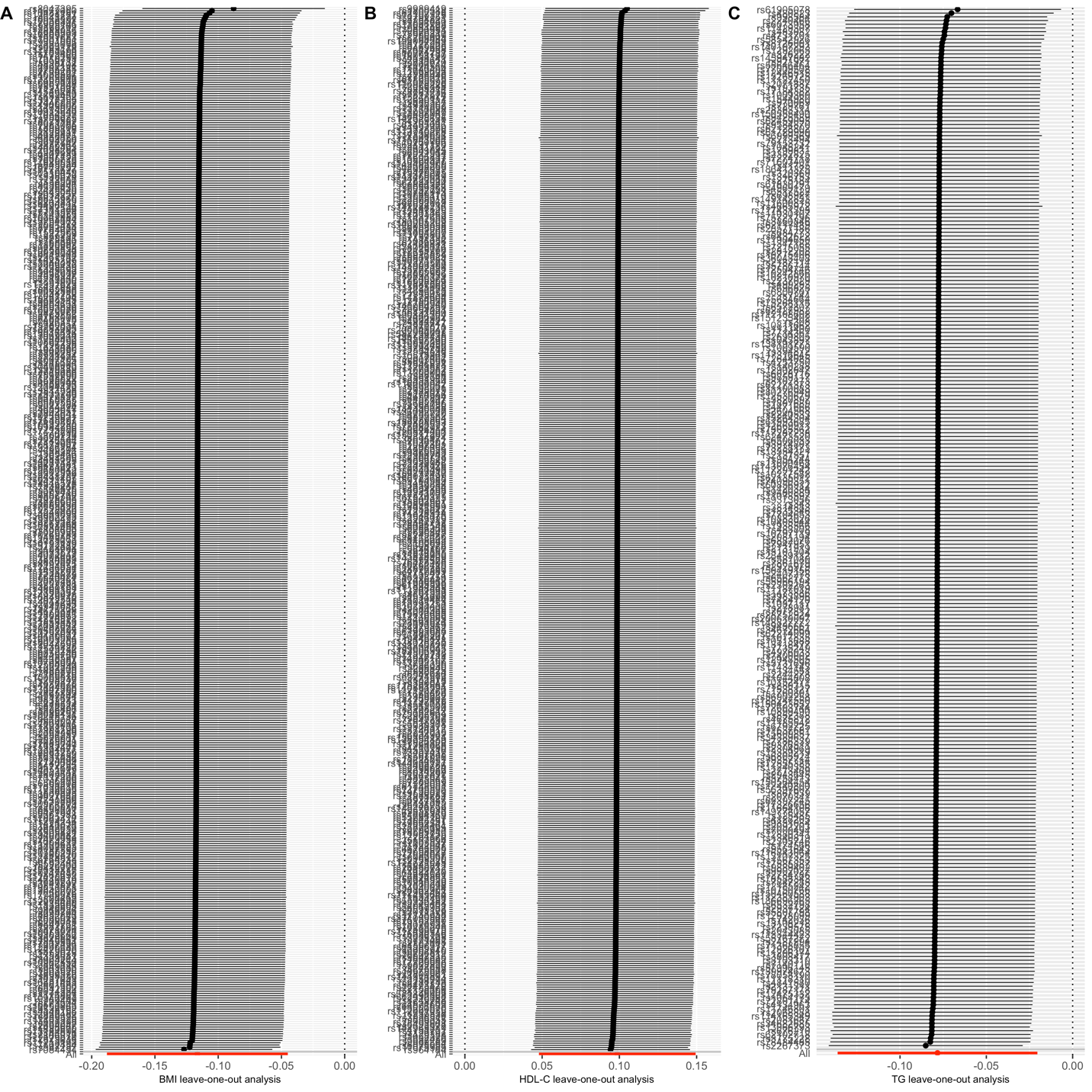


**Figure S9: Leave-one-out plot for BMI, HDL-C and TG with ER- negative breast cancer risk: (a) Height, (b) HDL-C, (c) TG.**  Plot shows the estimate effect by sequentially dropping one SNP at a time. Each black dot in the forest plot represents the MR results (IVW method) excluding that particular SNP. The result including all SNPs is shown in red at the bottom of the plot.


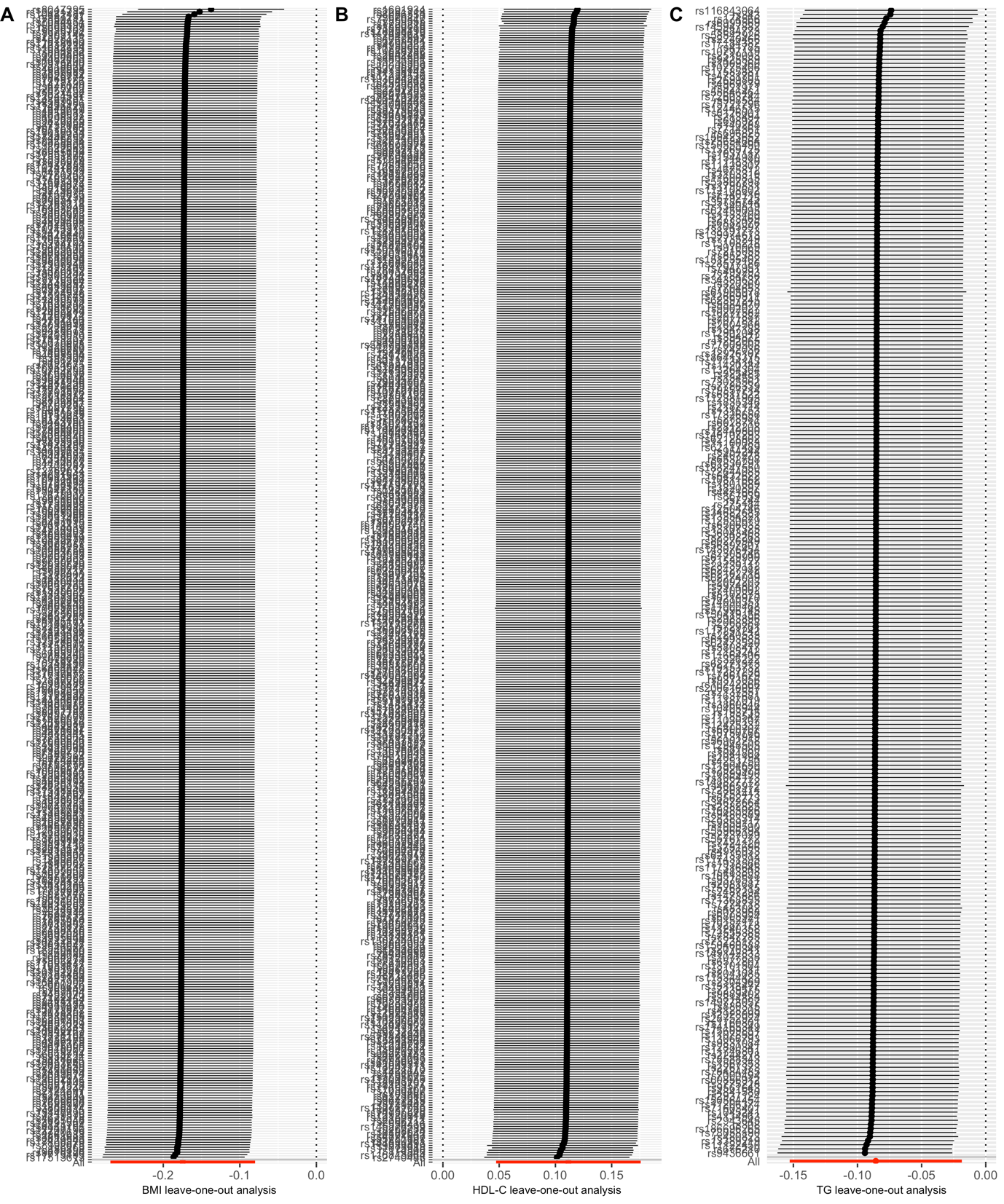

Supplement: Supplementary file 1 — Supplementary Information 1. [file 41598_2023_41130_MOESM1_ESM.docx]
